# The influence of emotional feedback material type on attentional capture at different presentation times

**DOI:** 10.1371/journal.pone.0310022

**Published:** 2024-09-16

**Authors:** Jiacheng Gao, Lin Jia, Suxia Wen, Yadi Jia, Guangxin Li, Hongli Liu

**Affiliations:** 1 Xinjiang Key Laboratory of Mental Development and Learning Science, School of Psychology, Xinjiang Normal University, Urumqi, Xinjiang, China; 2 Department of Psychology, Fudan University, Shanghai, China; 3 CAS Key Laboratory of Mental Health, Institute of Psychology, Beijing, China; 4 School of Educational Science, Xinjiang Normal University, Urumqi, Xinjiang, China; East Carolina University, CANADA

## Abstract

**Objective:**

This study aimed to explore the influence of emotional feedback materials on attentional capture at different presentation times and to investigate the mechanisms of positive and negative attentional biases.

**Methods:**

Two experiments were conducted. Experiment 1 recruited 47 participants, and Experiment 2 recruited 46 participants. Emotional facial images and emotional words were used as feedback materials. A learning-testing paradigm was employed to explore the effect of emotional feedback materials on attentional capture at different presentation times (1000 ms/100 ms).

**Results:**

We compared the accuracy and reaction times of participants under emotional and neutral conditions at both presentation times. Experiment 1 revealed that participants exhibited a stable positive attentional bias towards emotional facial images. Additionally, under the 100 ms feedback condition, emotional interference on judgment task accuracy was greater than under the 1000 ms feedback condition. Experiment 2 found that under the 100 ms feedback condition, emotional interference on reaction time was greater than under the 1000 ms feedback condition. Comparing the data from both experiments revealed that the processing time for emotional facial images was longer than for emotional words.

**Conclusions:**

(1) Emotional facial images are more effective than emotional words in capturing attention. (2) When positive and negative information with equal arousal levels alternates over a period of time, individuals exhibit a stable positive attentional bias. (3) When there is intense competition for attention and cognitive resources, emotional information is prioritized for processing.

## Introduction

Attentional capture is a crucial cognitive function. It is defined as the process of a novel stimulus captures attention unconstrained by the current task [1,2]. It includes goal-directed, stimulus-driven, and value-driven attentional capture. Previous research has underscored the pivotal role of attentional capture in shaping human perception and controlling behavior [3–7]. Due to the inherent conflict between the limited capacity of the sensory system and the vast information in the real world, individuals must use selective attention to process the most pertinent information for the current task, ensuring effective interaction with the environment [8,9]. However, emotionally salient stimuli possess a privileged status, as they can capture attention and be processed without undergoing the selective attention filter [10–12]. Moreover, emotional stimuli, when related to target stimuli as feedback, can guide attentional selection [13]. Once the association between target stimuli and emotional feedback is established, these previously feedback-related targets will automatically capture attention. From this perspective, previous studies have mostly focused on whether emotions affect attentional capture. However, substantial controversy exists among studies using different materials, presentation styles, and research paradigms regarding the role of emotions in attentional capture interference. To comprehensively understand the influence of emotion on attentional capture, it is crucial to clarify the role of emotional distractors. This study aimed to explore the influence of two types of emotional feedback materials on attentional capture and the roles of positive and negative emotional biases under different presentation time conditions.

The attentional bias hypothesis has been proposed to explain the impact of emotions on attentional capture [14]. When multiple informational stimuli compete for an individual’s attentional resources, a preference for positive information is termed positive attentional bias, while a preference for negative information is termed negative attentional bias [15,16]. Xu, He [17] systematically analyzed the evidence supporting both effects and found that when emotional faces were used as feedback material, factors such as the arousal level of the feedback material [18], gender [19,20], the individual’s cognitive processing style [21], and the presentation time [22] could all influence an individual’s attentional bias. This study attempted to clarify the roles of emotional feedback materials and presentation times in attentional capture.

Many previous researches on attentional capture have focused on confirming its existence and the presence of attentional bias [23–25]. For instance, Carretié, Mercado [26] explored the relationship between emotion and attentional bias using emotional images, finding that negative emotional stimuli elicited higher P200 component amplitudes and shorter latencies compared to positive stimuli. Similarly, an eye-tracking study revealed that participants experienced less distraction before attending to negative facial target stimuli, and their first fixation was more likely to land on negative facial target stimuli [27]. These studies confirm the presence of negative attentional bias in emotional information capture, supported by other experimental research [12,28–31]. Conversely, research using emotional Stroop tasks has found that under low time pressure conditions, positive emotional information captures attention [32]. Additional studies have validated the stable existence of positive attentional bias under various experimental conditions [33–35]. Pool, Brosch [16] reviewed previous research on attentional biases for emotional stimuli and pointed out that negative attentional bias is often associated with threat and anxiety and can induce attentional bias in both early and late stages of attention processing. In contrast, positive attentional bias is related to an individual’s level of emotional arousal and specific concerns (e.g., hunger, sexual attraction) and is more likely to occur in the early stages of attention processing. However, as research has progressed, several unresolved issues remain, particularly regarding the competition of attentional biases and the interfering role of presentation time [14,36–38].

Although studies on the influence of stimulus presentation time on attentional biases have focused on the impact of negative stimuli on special populations, they offer intriguing inferences. Research comparing depressed patients and healthy individuals under emotional picture presentation times of 100ms and 500ms found that, under the 100ms condition, depressed patients showed significantly weaker N1 and P1 component responses to negative pictures compared to the healthy control group. However, under the 500ms condition, depressed patients exhibited significantly higher P1 component amplitudes in response to negative pictures than healthy controls [39]. This indicates that, compared to healthy individuals, depressed patients are more likely to exhibit a negative attentional bias under longer presentation times. Moussally, Brosch [40] examined attentional biases toward body-related words in females at presentation times of 100ms, 500ms, and 1500ms and found similar results. Specifically, longer presentation times more easily triggered attentional biases in females dissatisfied with their bodies, with these biases being particularly evident in the N1 and P1 components. Recent studies on post-traumatic stress disorder (PTSD) patients also support these findings [41,42]. Conversely, a study investigating non-clinical anxious individuals’ attentional biases towards threatening words at presentation times ranging from 100ms to 1500ms found that highly anxious individuals exhibited attentional biases towards threatening stimuli at all presentation times. In contrast, low-anxiety individuals showed no significant attentional biases at any presentation time [43]. These studies suggest that, generally, the attentional bias toward emotional information in healthy individuals varies with stimulus material, individual state, and presentation time. Research indicates systematic differences when processing emotional words and images (especially faces) [44,45]. Therefore, verifying the differences in the impact of emotional words and emotional face images on attentional biases can help clarify the current controversy regarding the effects of emotional information on attentional biases.

The purpose of this study was to bridge the gap in the literature by designing experiments using emotional facial images and emotional words to investigate the influence of positive and negative emotional feedback on attentional capture, as well as the roles of positive and negative attentional biases under different presentation time conditions. To address this, we employed a learning-testing paradigm [35,46] to compare the impact of these two types of emotional feedback materials on attentional capture at different presentation times. Previous research has shown that when an individual’s selection of a target is consistently accompanied by a reward, the individual is more likely to choose that target stimulus [47]. Sali, Anderson [48] used a learning-testing paradigm to investigate how the reward prediction mechanism captures attention. This paradigm consists of two stages: learning and testing. The learning stage aims to establish a color-reward prediction mechanism for participants. Participants are required to identify a color-defined target in a search array containing six colored circles, with each correct identification resulting in a monetary reward. The testing stage is designed to verify whether the target-reward association established during the learning phase can interfere with attentional capture in the current task. In this stage, participants perform a second search task without rewards, where the target color is irrelevant, and the previously reward-associated color appears as a distractor. Participants must ignore the distractor color and search for a shape-defined target. Results showed that stimuli previously associated with rewards, particularly those linked to high rewards, could automatically capture attention. Some studies have explored whether emotional feedback stimuli can similarly influence attentional capture within the learning-testing paradigm. Izuma, Saito [49] used a learning-testing paradigm to compare the processing mechanisms of social and monetary rewards, finding that positive emotions and monetary rewards have similar mechanisms in capturing attention. Further studies using the learning-testing paradigm to explore the effects of positive and neutral facial emotional feedback on attentional capture found that only high-reward target colors (positive emotional faces) interfered with attentional capture during the testing phase [13]. These findings suggest that positive emotions can, through a simple associative learning process (learning phase), modulate individuals’ attention bias toward related stimuli during the testing phase. Some studies have also found that negative emotions affect attentional capture [50,51].

Based on the limited research, we have two hypotheses. First, because images convey more profound emotional information, we hypothesize that both emotional feedback materials capture attention, but emotional facial images are more effective in capturing attention than emotional words (Hypothesis 1). Second, previous studies have shown that the approximate onset and offset times for recognizing and processing emotional images and words range from 175ms to 800ms [23,52]. Stimuli with ecological significance, such as emotional content, are prioritized in attentional processing [11,53]. Therefore, we hypothesize that as the presentation time of feedback stimuli decreases, the attention bias towards emotional information will be strengthened. Compared to the neutral condition, at 100ms (insufficient processing), due to the competition for attentional resources, participants are more likely to exhibit an attentional bias toward emotional information. At 1000ms (sufficient processing), as the competition for attentional resources decreases, the attentional bias toward emotional information also diminishes (Hypothesis 2).

## Experiment 1: The influence of emotional word feedback materials on attentional capture at different presentation times

### Materials and methods

#### Participants

We conducted a power analysis using G*Power 3.1 [54]. Based on previous research and our experimental design, we set a medium effect size (*f* = 0.25), a significance level (*α* = 0.05), and a statistical power (power = 0.95). The calculation indicated that at least 44 participants were needed to detect the expected effect. Ultimately, through online poster advertisements, 47 participants were recruited for Experiment 1 (15 males and 32 females). The participants had a mean age of 20.49 ± 2.00 years, with ages ranging from 19 to 32 years. All participants had normal or corrected visual acuity. Each participant received a small gift (worth 15 RMB) at the end of the experiment. The participants voluntarily took part in the experiment and signed written informed consent forms. The study was approved by the Ethics Review Committee of the School of Psychology at Xinjiang Normal University (XJNUPSY-2023-05). The experiment was conducted from May 13 to June 15, 2024.

#### Experimental design

The experiment employed a 2 (Feedback Time: 1000ms vs. 100ms) × 3 (Interference Condition: Positive Emotional Interference vs. Negative Emotional Interference vs. No Interference) two-factor design. Feedback time was a between-participants variable, while interference condition was a within-participants variable. The dependent variables were participants’ reaction times and accuracy rates.

#### Stimuli and apparatus

The experimental materials were selected from the Chinese Affective Words System (CAWS) [55]. According to the CAWS manual, both valence and arousal are scored on a 9-point scale. The lower the score for valence or arousal, the closer it is to 1; the higher the score, the closer it is to 9. Based on the CAWS evaluation data, we matched the arousal levels and selected 40 positive, 40 negative, and 40 neutral Chinese 2-character words. The valence differences among the three groups of words were significant [*F*_(2, 117)_ = 565.88, *p* < 0.001], while the arousal differences were not significant [*F*_(2, 117)_ = 0.01, *p* = 0.99]. The selected words are listed in the (S1 File). Information on the valence and arousal of the words is shown in Table 1.

**Table 1:** Valence and arousal of affective words.

Word Type	Valence		Arousal degree	
	*M*	*SD*	*M*	*SD*
**Positive Words**	6.57	0.27	4.69	0.13
**Neutral Words**	5.31	0.55	4.69	0.31
**Negative Words**	3.44	0.38	4.68	0.42

The experimental stimuli were presented on a 21-inch Lenovo Yangtian desktop computer with a screen resolution of 1024 × 768. The experiment was programmed using E-Prime 2.0. The participants were seated approximately 55 cm away from the screen.

#### Procedure

**Fig 1:**
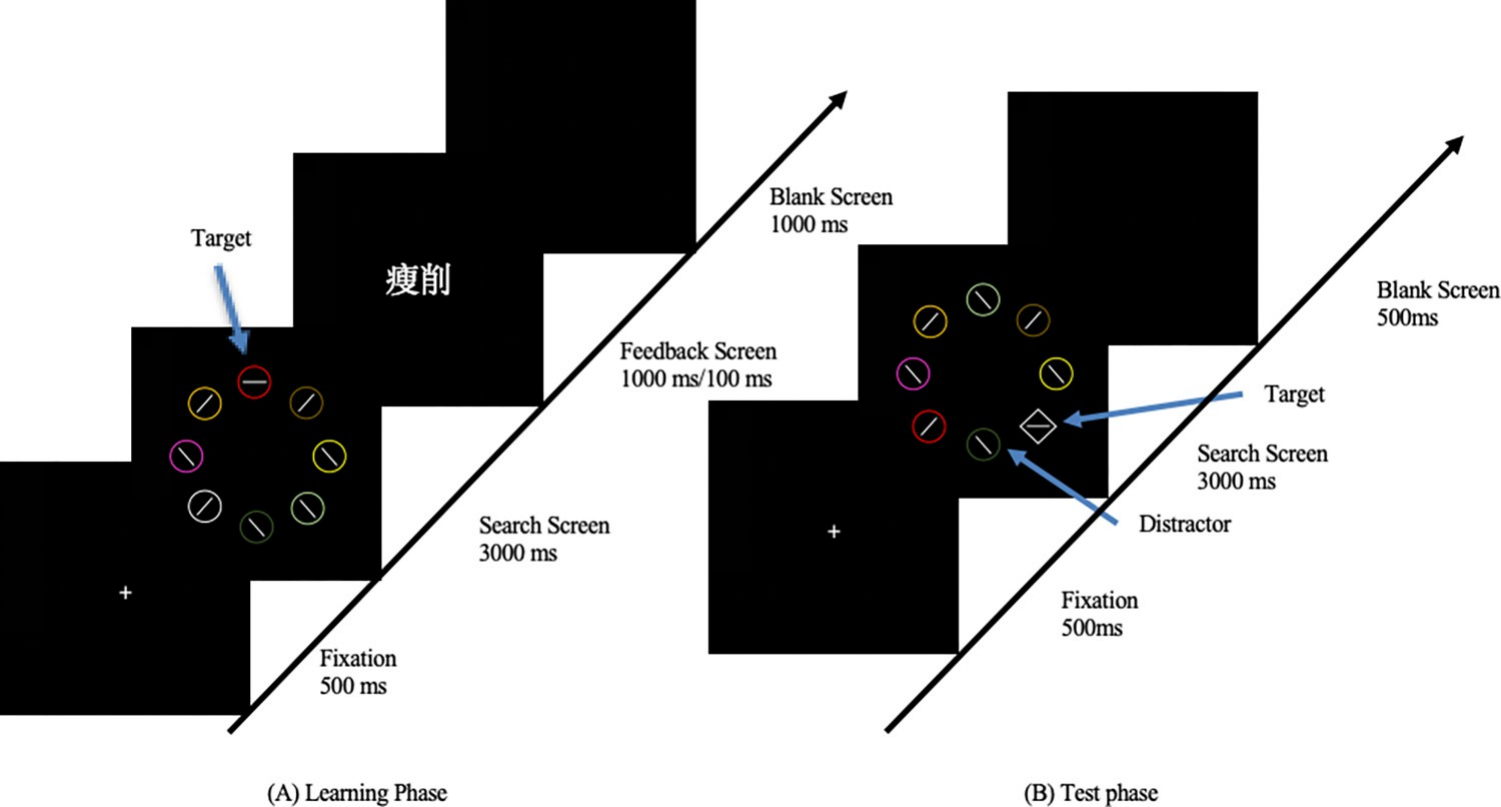
Experimental process.

The experimental procedures referred to the learning-testing paradigm [135]. The learning phase aims to establish a strong association between colors and target emotions (positive and negative). The testing phase was designed to verify whether the previously established strong associations can interfere with attention capture during the current search task, specifically assessing whether there is an attentional bias toward emotional information. During the learning phase, each trial consisted of four parts (see Fig 1A). First, a white fixation point "+" was presented at the center of the screen for 500 ms. Next, the search phase began, where the screen displayed a virtual large circle composed of eight smaller circles of different colors. One of these was the target circle (red, blue, or cyan), and the other seven were distractor circles of various colors (yellow, brown, light green, dark green, pink, purple, white). Each small circle contained a white line segment. The line segment inside the target circle was either horizontal or vertical, while the line segments in the distractor circles were tilted 45° to the left or right. Participants were required to determine the orientation of the line segment in the target circle within 3000 ms and respond by pressing a key. Following this, the feedback phase ensued. Regardless of whether the participant’s judgment was correct, positive emotional words were provided as feedback in the red target circle trial, negative emotional words in the blue target circle trial, and neutral words in the cyan target circle trial. This feedback aimed to establish an association between color and emotional feedback. The emotional feedback words were selected from the CAWS [55]. Participants were randomly assigned to either a 1000 ms feedback time group or a 100 ms presentation time group. After the feedback phase, a blank screen was shown for 1000 ms before the next trial began. The entire learning phase consisted of 10 practice trials with accuracy feedback and 360 formal trials. To test the association between color and emotion, we set the positive and negative emotion feedback as a strong association and presented it in 162 trials each, for a total of 324 trials (90%). Neutral feedback was set as a weak association and presented 36 times (10%). To balance participants’ familiarity with the emotional words, the selection of emotional words employed random sampling with replacement.

Each trial in the testing phase consisted of three parts (see Fig 1B). First, a white fixation point "+" appeared on the screen for 500 ms. Then, the search phase began. The screen displayed a virtual large circle composed of seven smaller circles of different colors and one smaller diamond. The diamond was the target stimulus and was a color other than red, blue, or cyan. The color of the distractor circles varied according to the interference conditions: no interference (excluding red, blue, and cyan circles), positive emotional interference (including one red circle), and negative emotional interference (including one blue circle). The search phase lasted for 3000 ms. During this phase, participants were instructed to ignore the color of the diamond and only judge the orientation of the line segment inside it. The response rules were the same as in the learning phase. Each interference condition appeared 60 trials, with the order of presentation randomized. Finally, a blank screen was shown for 500 ms before the next trial began. The testing phase included 10 practice trials with accuracy feedback and 180 formal trials.

#### Statistical analyses

We used SPSS 26 for data analysis. A 2 (Feedback Time: 1000 ms vs. 100 ms) × 2 (Association Type: Strong vs. Weak) mixed ANOVA was conducted on reaction times and accuracy during the learning phase to assess the establishment of color-emotion associations. For the testing phase, a 2 (Feedback Time: 1000 ms vs. 100 ms) × 3 (Interference Condition: Positive Emotion Interference vs. Negative Emotion Interference vs. No Interference) mixed ANOVA was performed on reaction times and accuracy. This analysis aimed to evaluate the impact of different emotions on attentional bias at different feedback times.

## Results

Data from participants with an accuracy rate below 80% were excluded, resulting in a removal rate of 6.38% (3 participants). The analysis was conducted on reaction time and accuracy separately.

### Learning phase

**Fig 2:**
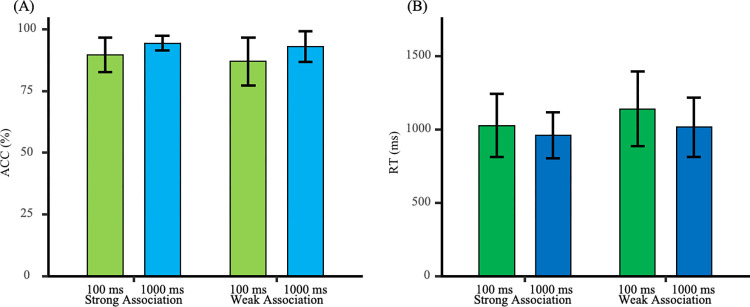
Reaction times and accuracy in the learning phase. (A) Reaction times for strongly associated stimuli were significantly shorter than those for weakly associated stimuli. (B) Accuracy for strongly associated stimuli was significantly higher than that for weakly associated stimuli.

Reaction times and accuracy rates during the learning phase are presented in Fig 2. A 2 (Feedback Time: 1000 ms vs. 100 ms) × 2 (Association Type: Strong vs. Weak) mixed ANOVA was conducted to examine the establishment of color-emotion associations.

#### Reaction times

The main effect of association type was significant [*F*_(1, 42)_ = 15.28, *p* < 0.001, *η*_p_^2^ = 0.27]. The main effect of feedback time was not significant [*F*_(1, 42)_ = 2.86, *p* = 0.10, *η*_p_^2^ = 0.06]. The interaction effect was not significant [*F*_(1, 42)_ = 0.35, *p* = 0.56, *η*_p_^2^ = 0.01]. Further analysis revealed that reaction times for strong association stimuli were significantly lower than for weak association stimuli [*t*_(43)_ = −3.94, *p* < 0.001, Cohen’s *d* = 0.59]. This indicates that strong association trials established a tighter color-emotion link during the learning phase.

#### Accuracy

The main effect of association type was significant [*F*_(1, 42)_ = 9.20, *p* = 0.004, *η*_p_^2^ = 0.18]. The main effect of feedback time was not significant [*F*_(1, 42)_ = 3.22, *p* = 0.08, *η*_p_^2^ = 0.07]. The interaction effect was not significant [*F*_(1, 42)_ = 1.05, *p* = 0.31, *η*_p_^2^ = 0.02]. Further analysis showed that accuracy for strong association stimuli was significantly higher than for weak association stimuli [*t*_(43)_ = 3.03, *p* = 0.004, Cohen’s *d* = 0.46]. This also suggests that strong association trials established a tighter color-emotion link during the learning phase.

### Testing phase

**Fig 3:**
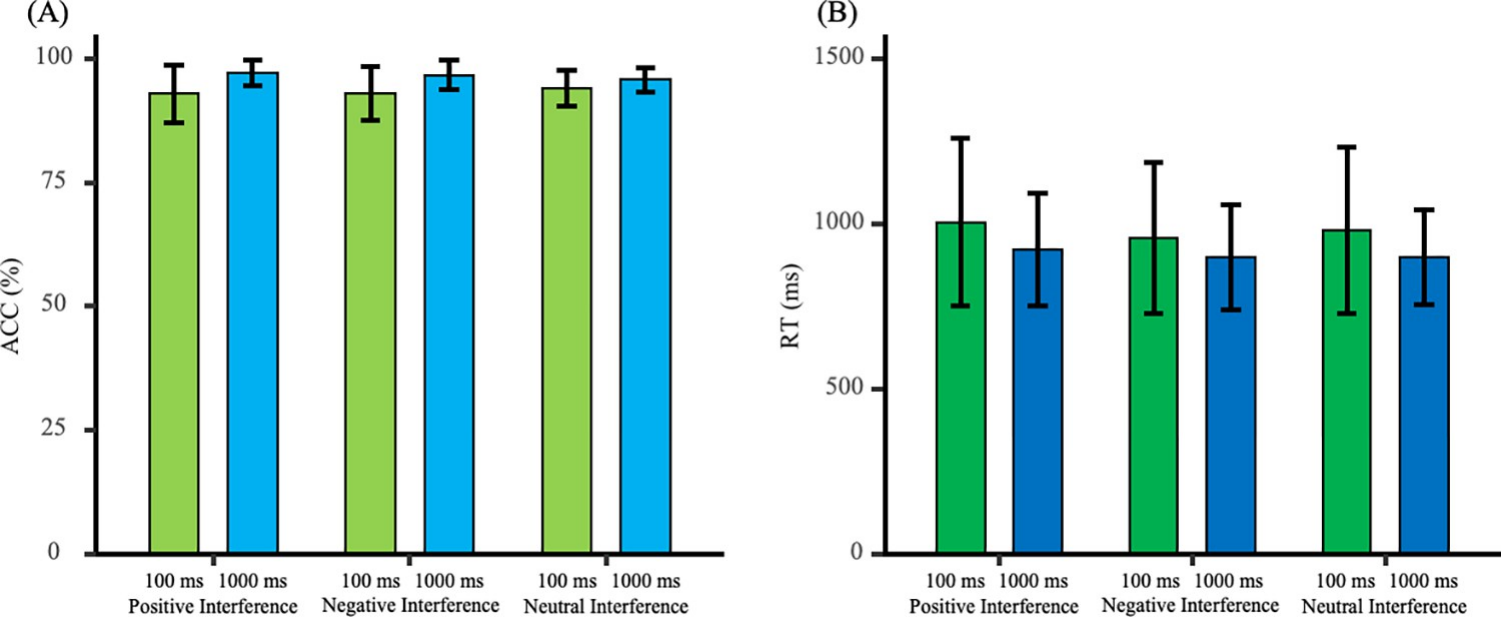
Reaction times and accuracy in the testing phase. (A) Reaction times under the 1000 ms feedback condition were shorter than those under the 100 ms condition.

Reaction times and accuracy rates during the testing phase are presented in Fig 3. A 2 (Feedback Time: 1000 ms vs. 100 ms) × 3 (Interference Condition: Positive Emotion Interference vs. Negative Emotion Interference vs. No Interference) mixed ANOVA was conducted to examine the effect of emotional interference on attentional bias under different feedback times.

#### Reaction times

The main effect of interference type was not significant [*F*_(2, 84)_ = 2.61, *p* = 0.08, *η*_p_^2^ = 0.06]. The main effect of feedback time was significant [*F*_(1, 42)_ = 4.44, *p* = 0.04, *η*_p_^2^ = 0.10]. The interaction effect was not significant [*F*_(2, 84)_ = 0.58, *p* = 0.56, *η*_p_^2^ = 0.01]. Further analysis indicated that reaction times were lower under the 1000 ms feedback time compared to the 100 ms condition [*t*_(42)_ = −2.11, *p* = 0.04, Cohen’s *d* = 0.64]. This suggests that emotional word interference was greater under the 100 ms feedback time condition.

#### Accuracy

The main effect of interference type was not significant [*F*_(2, 84)_ = 0.94, *p* = 0.40, *η*_p_^2^ = 0.02]. The main effect of feedback time was not significant [*F*_(1, 42)_ = 0.33, *p* = 0.57, *η*_p_^2^ = 0.01]. The interaction effect was not significant [*F*_(2, 84)_ = 0.96, *p* = 0.39, *η*_p_^2^ = 0.02].

These results suggest that feedback time influences the effect of emotional interference on attentional bias, with shorter feedback times leading to greater interference from emotional words.

## Experiment 2: The influence of emotional facial images feedback materials on attentional capture at different presentation times

### Materials and methods

#### Participants

A power analysis for sample size was conducted similar to Experiment 1. In Experiment 2, 46 participants were recruited (15 males and 31 females) through online poster advertisements,. The participants had a mean age of 20.15 ± 1.63 years, with ages ranging from 18 to 26 years. All participants had normal or corrected visual acuity. Each participant received a small gift (worth 15 RMB) at the end of the experiment. The participants voluntarily took part in the experiment and signed written informed consent forms. The experiment was conducted for the period May 13 to June 11, 2024.

#### Experimental design

Same as Experiment 1.

#### Stimuli and apparatus

Experimental materials were selected from the revised Chinese Facial Affective Picture System (CFAPS) [56]. According to the CFAPS manual, both pleasantness and arousal are scored on a 9-point scale. The lower the score for pleasantness or arousal, the closer it is to 1; the higher the score, the closer it is to 9. Based on the CFAPS evaluation data, we matched the arousal levels and selected 40 positive facial expressions, 40 negative facial expressions, and 40 neutral facial expressions, with an equal distribution between genders. As shown in Table 2, the three types of emotional facial images differed significantly in pleasantness [*F*_(2, 117)_ = 161.19, *p* < 0.001], while there was no significant difference in arousal [*F*_(2, 117)_ = 0.03, *p* = 0.97]. The selected facial expressions are displayed in the (S1 File).

**Table 2:** Pleasantness and arousal of emotional facial images.

Picture Type	Pleasantness		Arousal degree	
	*M*	*SD*	*M*	*SD*
**Positive Face**	5.72	0.68	4.29	0.68
**Neutral Face**	4.32	0.57	4.28	0.67
**Negative Face**	3.40	0.48	4.26	0.66

The experimental apparatus was the same as in Experiment 1.

#### Procedures

Same as Experiment 1.

#### Statistical analyses

Same as Experiment 1.

## Results

Data from participants with an accuracy rate below 80% were excluded, resulting in a removal rate of 4.35% (2 participants). Reaction time and accuracy were analyzed separately.

### Learning phase

**Fig 4:**
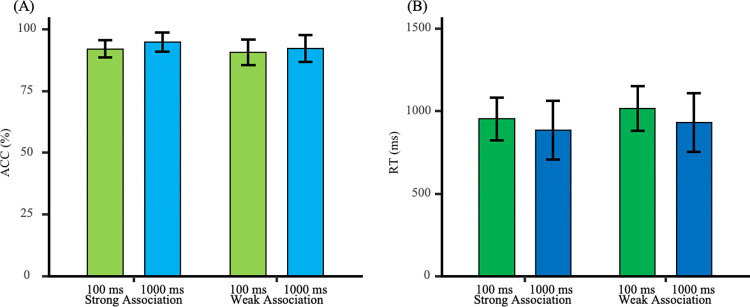
Reaction times and accuracy rates during the learning phase. (A) Reaction times for strongly associated stimuli were significantly shorter than those for weakly associated stimuli. (B) Accuracy under the 1000 ms feedback condition was higher than that under the 100 ms feedback condition.

Reaction times and accuracy rates during the learning phase are presented in Fig 4. A 2 (Feedback Time: 1000 ms vs. 100 ms) × 2 (Association Type: Strong vs. Weak) mixed ANOVA was conducted to examine the establishment of color-emotion associations.

#### Reaction times

The main effect of association type was significant [*F*_(1, 42)_ = 19.04, *p* < 0.001, *η*_p_^2^ = 0.31]. The main effect of feedback time was not significant [*F*_(1, 42)_ = 2.53, *p* = 0.12, *η*_p_^2^ = 0.06]. The interaction effect was not significant [*F*_(1, 42)_ = 2.19, *p* = 0.15, *η*_p_^2^ = 0.05]. Further analysis showed that reaction times for strongly associated stimuli were significantly shorter than for weakly associated stimuli [*t*_(43)_ = -4.30, *p* < 0.001, Cohen’s *d* = 0.82]. This indicates that strong associations between color and emotion were more firmly established during the learning phase.

#### Accuracy

The main effect of association type was not significant [*F*_(1, 42)_ = 3.14, *p* = 0.08, *η*_p_^2^ = 0.07]. The main effect of feedback time was significant [*F*_(1, 42)_ = 9.87, *p* = 0.003, *η*_p_^2^ = 0.19]. The interaction effect was not significant [*F*_(1, 42)_ = 0.30, *p* = 0.58, *η*_p_^2^ = 0.01]. Further analysis indicated that the accuracy rate under the 1000 ms feedback time was higher than under the 100 ms feedback time [*t*_(42)_ = 3.14, *p* = 0.003, Cohen’s *d* = 0.95]. This suggests that emotional facial feedback images have a greater impact on the judgment task at 100 ms feedback time.

### Testing phase

**Fig 5:**
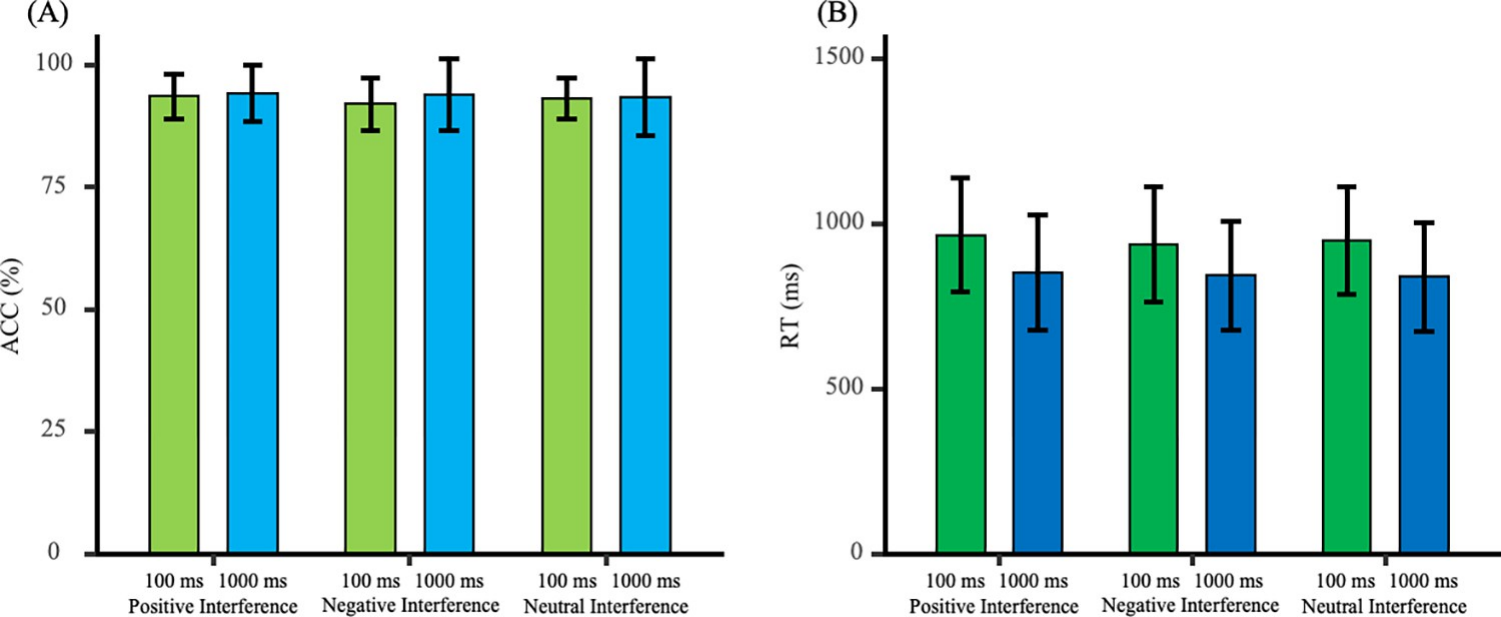
Reaction times and accuracy rates during the testing phase. (A) Reaction times under the positive interference condition were higher than those under the no interference and negative interference conditions, with no difference in reaction times between the negative interference and no interference conditions. (B) Accuracy under the 1000 ms feedback condition was higher than that under the 100 ms feedback condition.

The reaction times and accuracy rates during the testing phase are shown in Fig 5. A 2 (Feedback Time: 1000 ms vs. 100 ms) × 3 (Interference Condition: Positive Emotion Interference vs. Negative Emotion Interference vs. No Interference) mixed ANOVA was conducted to examine the effect of emotional interference on attentional bias at different feedback times.

#### Reaction times

The main effect of feedback time was not significant [*F*_(1, 42)_ = 1.44, *p* = 0.24, *η*_p_^2^ = 0.03]. The main effect of interference condition was significant [*F*_(2, 84)_ = 7.64, *p* = 0.001, *η*_p_^2^ = 0.15]. The interaction effect was not significant [*F*_(2, 84)_ = 1.00, *p* = 0.37, *η*_p_^2^ = 0.02]. Further analysis revealed that reaction times under positive interference were longer than those under negative interference [*t*_(43)_ = 3.72, *p* = 0.001, Cohen’s *d* = 0.56] and longer than those under no interference [*t*_(43)_ = 2.53, *p* = 0.02, Cohen’s *d* = 0.38]. There was no significant difference between negative interference and no interference [*t*_(43)_ = -1.28, *p* = 0.21, Cohen’s *d* = 0.19]. This suggests a consistent positive attention bias under both feedback time conditions.

#### Accuracy

The main effect of feedback time was significant [*F*_(1, 42)_ = 9.75, *p* = 0.003, *η*_p_^2^ = 0.19]. The main effect of interference condition was not significant [*F*_(2, 84)_ = 0.04, *p* = 0.96, *η*_p_^2^ = 0.001]. The interaction effect was not significant [*F*_(2, 84)_ = 2.59, *p* = 0.08, *η*_p_^2^ = 0.06]. Further analysis showed that the accuracy rate under the 1000 ms feedback condition was higher than under the 100 ms feedback condition [*t*_(42)_ = 3.12, *p* = 0.003, Cohen’s *d* = 0.94]. This indicates that emotional facial images had a greater interference effect on judgment tasks under the 100 ms feedback time.

### Comparison between emotional facial images and words

To further compare the effects of emotional facial images feedback and emotional word feedback, data from Experiment 1 and Experiment 2 were combined for analysis.

#### Learning phase

A 2 (Emotional Material: Faces vs. Words) × 2 (Feedback Time: 1000ms vs. 100ms) × 2 (Association Type: Strong vs. Weak) mixed ANOVA was conducted to examine the establishment of associations between color and emotion using different emotional materials.

*Reaction times*. The main effect of association type was significant [*F*_(1, 84)_ = 33.87, *p* < 0.001, *η*_p_^2^ = 0.29]. The main effect of feedback time was significant [*F*_(1, 84)_ = 5.23, *p* = 0.03, *η*_p_^2^ = 0.06]. The main effect of emotional material was significant [*F*_(1, 84)_ = 5.70, *p* = 0.02, *η*_p_^2^ = 0.06]. The interaction between association type and feedback time was not significant [*F*_(1, 84)_ = 2.40, *p* = 0.13, *η*_p_^2^ = 0.03]. The interaction between association type and emotional material was not significant [*F*_(1, 84)_ = 1.68, *p* = 0.20, *η*_p_^2^ = 0.02]. The interaction among association type, feedback time, and emotional material was not significant [*F*_(1, 84)_ = 0.75, *p* = 0.39, *η*_p_^2^ = 0.01]. The interaction between feedback time and emotional material was not significant [*F*_(1, 84)_ = 0.07, *p* = 0.79, *η*_p_^2^ = 0.001].

Further analysis of reaction times for different association types showed that reaction times for strong association stimuli were significantly shorter than for weak association stimuli [*t*_(87)_ = -5.76, *p* < 0.001, Cohen’s *d* = 0.62]. This indicates that both emotional facial images and emotional words can establish color-emotion associations. Further analysis of reaction times for different feedback times showed that reaction times under the 1000ms feedback time were faster than under the 100ms condition [*t*_(86)_ = -2.24, *p* = 0.03, Cohen’s *d* = 0.48], indicating that emotional feedback materials have a greater impact on judgment tasks under the 100ms feedback time. Further analysis of reaction times for different emotional materials showed that reaction times for facial images were higher than for words [*t*_(86)_ = 2.34, *p* = 0.02, Cohen’s *d* = 0.50], suggesting that emotional facial materials have a greater impact on the judgment task during the association establishment process.

*Accuracy*. The main effect of association type was significant [*F*_(1, 84)_ = 9.11, *p* = 0.003, *η*_p_^2^ = 0.10]. The main effect of feedback time was significant [*F*_(1, 84)_ = 12.96, *p* = 0.001, *η*_p_^2^ = 0.13]. The main effect of emotional material was not significant [*F*_(1, 84)_ = 1.94, *p* = 0.17, *η*_p_^2^ = 0.02]. The interaction between association type and feedback time was not significant [*F*_(1, 84)_ = 0.00, *p* = 0.99, *η*_p_^2^ = 0.00]. The interaction between association type and emotional material was not significant [*F*_(1, 84)_ = 0.01, *p* = 0.94, *η*_p_^2^ = 0.00]. The interaction among association type, feedback time, and emotional material was not significant [*F*_(1, 84)_ = 0.96, *p* = 0.33, *η*_p_^2^ = 0.01]. The interaction between feedback time and emotional material was not significant [*F*_(1, 84)_ = 2.30, *p* = 0.13, *η*_p_^2^ = 0.03].

Further analysis of accuracy rates for different association types showed that accuracy rates for strong association stimuli were significantly higher than for weak association stimuli [*t*_(87)_ = 3.05, *p* = 0.003, Cohen’s *d* = 0.33]. This indicates that both emotional facial images and emotional words can establish color-emotion associations. Further analysis of accuracy rates for different feedback times showed that accuracy rates under the 1000ms feedback time were higher than under the 100ms condition [*t*_(86)_ = 3.55, *p* = 0.001, Cohen’s *d* = 0.76], indicating that emotional feedback materials have a greater impact on judgment tasks under the 100ms feedback time.

#### Testing phase

The reaction times and accuracy rates during the testing phase were analyzed using a 2 (Emotional Material: Faces vs. Words) × 2 (Feedback Time: 1000ms vs. 100ms) × 3 (Interference Condition: Positive vs. Negative vs. Neutral) mixed ANOVA to examine the influence of different emotional materials on participants’ attention bias.

*Reaction Times*. The main effect of interference condition was significant [*F*_(2, 168)_ = 9.71, *p* < 0.001, *η*_p_^2^ = 0.10]. The main effect of feedback time was significant [*F*_(1, 84)_ = 5.11, *p* = 0.03, *η*_p_^2^ = 0.06]. The main effect of emotional material was not significant [*F*_(1, 84)_ = 1.35, *p* = 0.25, *η*_p_^2^ = 0.02]. The interaction between interference condition and feedback time was not significant [*F*_(2, 168)_ = 1.54, *p* = 0.22, *η*_p_^2^ = 0.02]. The interaction between interference condition and emotional material was not significant [*F*_(2, 168)_ = 0.88, *p* = 0.42, *η*_p_^2^ = 0.01]. The interaction among interference condition, feedback time, and emotional material was not significant [*F*_(2, 168)_ = 0.06, *p* = 0.94, *η*_p_^2^ = 0.001]. The interaction between feedback time and emotional material was not significant [*F*_(1, 84)_ = 0.16, *p* = 0.69, *η*_p_^2^ = 0.002].

Further analysis of reaction times for different interference conditions showed that reaction times under positive interference were higher than under negative interference [*t*(87) = 3.93, *p* < 0.001, Cohen’s *d* = 0.42], and higher than under no interference [*t*(87) = 3.08, *p* = 0.003, Cohen’s *d* = 0.33]. There was no significant difference between negative interference and no interference [*t*(87) = -1.29, *p* = 0.20, Cohen’s *d* = 0.14], indicating the stable presence of positive attention bias (H2). Further analysis of reaction times for different feedback times showed that reaction times under the 1000ms feedback time were lower than under the 100ms condition [*t*(86) = -2.26, *p* = 0.03, Cohen’s *d* = 0.48], indicating that emotional materials have a greater interference effect on judgment tasks under the 100ms feedback time.

*Accuracy*. The main effect of interference condition was not significant [*F*_(2, 168)_ = 0.75, *p* = 0.48, *η*_p_^2^ = 0.01]. The main effect of feedback time was significant [*F*_(1, 84)_ = 4.57, *p* = 0.04, *η*_p_^2^ = 0.05]. The main effect of emotional material was not significant [*F*_(1, 84)_ = 2.40, *p* = 0.13, *η*_p_^2^ = 0.03]. The interaction between interference condition and feedback time was not significant [*F*_(2, 168)_ = 2.59, *p* = 0.08, *η*_p_^2^ = 0.03]. The interaction between interference condition and emotional material was not significant [*F*_(2, 168)_ = 0.36, *p* = 0.70, *η*_p_^2^ = 0.004]. The interaction among interference condition, feedback time, and emotional material was not significant [*F*_(2, 168)_ = 0.73, *p* = 0.48, *η*_p_^2^ = 0.01]. The interaction between feedback time and emotional material was not significant [*F*_(1, 84)_ = 1.36, *p* = 0.25, *η*_p_^2^ = 0.02].

Further analysis of accuracy rates for different feedback times showed that accuracy rates under the 1000ms condition were higher than under the 100ms condition [*t*_(86)_ = 2.12, *p* = 0.04, Cohen’s *d* = 0.45], indicating that emotional materials have a greater interference effect on judgment tasks under the 100ms feedback time.

## Discussion

Our experiment yielded three main findings. First, when emotional faces were used as feedback materials, they consistently elicited a positive attentional bias in individuals. Additionally, under the 100 ms feedback condition, emotional interference with task accuracy was greater. Second, unlike emotional faces, when emotional words were used as feedback materials, emotional interference with reaction time was greater under the 100 ms feedback condition. Third, combining the results of the two experiments, we found that the processing time for emotional faces was longer than that for emotional words.

### The influence of emotional facial images and emotional words on attention capture

This study demonstrates that both emotional facial images and emotional words have the capacity to capture attention, aligning with previous research findings [14,57]. However, it is important to recognize that these two types of stimuli capture attention through different mechanisms. Theoretical models of attention for emotional information suggest that the cognitive processing of emotional faces is distinctive due to their socially and physiologically relevant properties [58]. Emotional faces are processed by a dedicated system, the right Fusiform Face Area (FFA), which exclusively handles facial stimuli [59]. As a result, emotional face processing occurs in the pre-attentive stage and requires fewer attentional resources, making it more effective at capturing attention [60]. In contrast, according to the serial model of emotional word processing, the semantic processing of words occurs at least 200 ms after word presentation [61]. This study found that attention can also be captured at a presentation time of 100 ms for emotional words, indicating that individuals can process emotional words with earlier acquisition of emotional meaning than conceptual processing. At 100 ms, participants were already able to capture the emotional information conveyed by the word. This supports the associative learning mechanism of emotional word processing, where repeated associations between the visual image of a word and its emotional connotation allow readers to directly access the emotional lexical meaning from the visual features of the word, leading to an emotional impact [62]. Some brain function studies have also found differences in ERP components between neutral and emotional words at 100 ms, indicating that emotional influences are already present at this time [63]. Therefore, emotional words are less effective as feedback material compared to emotional facial images [64], and emotional words elicit weaker emotional effects than emotional facial images [63].

In addition, the characteristics of the two emotional feedback materials significantly influenced the experimental results. The expressions in the CFAPS were precisely classified and easily recognized. In contrast, the CAWS only distinguished between positive and negative emotion words, conveying relatively complex and uncontrollable emotional information. Different participants may experience different emotions when exposed to the same word, and a single term may evoke multiple emotions. For instance, although "praise" is a positive term, socially anxious individuals may also feel anxious when confronted with this word, suggesting that "praise" may evoke more than just positive emotions for them [65]. Therefore, while this study found that emotional words were less effective as feedback material compared to emotional facial images, this conclusion is limited to the specific materials used in this study. If the emotional words were more precisely categorized into different emotion types, or if the facial expressions were replaced with more complex expressions, the similarities and differences in attentional capture between emotional facial images and emotional words could be more reliably compared.

### Independent effects of emotional interference and feedback time on attention bias

Experiment 1 used emotional words as feedback material and found that emotional interference was greater at the 100ms feedback time compared to the 1000ms feedback time. Similarly, Experiment 2, which used emotional facial images as feedback material, also found greater emotional interference at the 100ms feedback time compared to the 1000ms feedback time. Additionally, Experiment 2 identified an independent effect of positive attentional bias. This could be because participants established both positive and negative emotional associations during the learning phase, with positive attention bias dominating. Previous research indicates that participants preferred more threatening stimuli when the stimulus presentation time in a dot-probe task was 100ms. However, when the presentation time was extended to 500ms, participants developed an attentional bias toward more positive stimuli [36]. Calvo, Nummenmaa [66] analyzed ERP components of participants viewing faces with various emotional expressions and found that the processing of negative emotional faces, such as anger, occurred in the early components and was automatic, while the processing of positive emotional faces appeared in the later components.

This reflects different strategies of emotion processing under conscious and unconscious conditions: under conscious conditions with sufficient time, individuals prefer to process positive emotional information, whereas under unconscious conditions with time constraints, individuals preferentially attend to negative emotional information. Our study shows different results. Participants exhibited a stable positive attentional bias under both feedback time conditions. While this seems to conflict with previous findings, it may clarify the post-attention processing bias mechanism from a behavioral experimental perspective. In other words, over a period of time, when positive and negative information of equal arousal levels competed for an individual’s attention and cognitive resources, the individual would preferentially process positive information.

An unexpected finding was that the two types of emotional materials interfered with attentional bias through different mechanisms. Emotional facial images used as feedback interfered with the accuracy of judgment tasks during the testing phase, whereas emotional words interfered with reaction times in these tasks. Our results indicate that emotional information has processing priority under unconscious or insufficient processing conditions (100ms). This priority diminishes under conscious or sufficient processing conditions (1000ms). This suggests that under unconscious conditions, emotional information is preferentially processed when there is competition for attention or cognitive resources. However, under sufficient processing conditions (1000ms), emotional information is fully processed, thereby reducing interference with the processing of other information, such as judgment tasks.

In our experiments, the processing priority for emotional information was fully manifested with emotional facial materials. Under the 100ms feedback time, attention and cognitive resources were preferentially allocated to processing facial emotional information during the learning phase. This strengthened the color-emotion association and interfered with the accuracy of judgment tasks in the testing phase. Conversely, under the 1000ms feedback time, the extended processing time for emotional faces reduced their interference with judgment tasks, as the competition for attention and cognitive resources decreased. This competition also occurred with word materials but manifested differently. During the word feedback phase, participants in the 100ms feedback condition had longer reaction times in judgment tasks. This puzzling result might be explained by the possibility that participants’ emotional interference paths in the testing phase were color-emotion-word rather than direct color-emotion. This aligns with the serial model of emotional word processing. Under the 100ms feedback condition, participants may have established a preferential color-emotion-word association processing strategy during the learning phase and continued using this strategy in the testing phase. Consequently, participants required additional time to process emotional interference through word processing. Furthermore, because words are less capable of capturing attention than faces, this interference did not manifest in accuracy rates. These different forms of interference confirm the existence of a dedicated processing system for emotional faces and a serial model for emotional word processing.

## Limitations and prospects

Emotion is a social stimulus, and laboratory studies often suffer from poor ecological validity. The influence of unfamiliar negative emotional faces in a controlled laboratory environment may not fully replicate the impact of emotional information encountered in daily life. Consequently, attentional biases towards emotional information in real-life situations might be more common and influential than those observed in this study. Future research could improve ecological validity by selecting images of familiar faces for participants or investigating the effect of emotional information in real-life situations on attentional bias. This study employed a learning-testing paradigm and found a stable positive attention bias when positive and negative emotional information with equal arousal levels were presented simultaneously. Our experimental design led to results differing from previous studies. Future research should consider setting up groups with exclusively positive or negative emotional associations or manipulating the arousal levels of emotional stimuli to verify and supplement our findings. Additionally, our experiment did not strictly balance gender across groups, potentially introducing bias into our results. Future studies should address this issue. Finally, comparing emotional materials across Eastern and Western cultures, especially between emotional faces and words, will help clarify the role of culture in emotional attention bias. Some studies suggest that the allocation of attention resources can reduce attention capture. In the present experiment, participants had to perform tasks such as judging the direction of the line segment, monitoring the color of the circle, keeping track of the assigned buttons, and processing the direction of the line segment, potentially leading to over-occupation of cognitive resources. Future studies may consider simplifying the cognitive load imposed on participants during the experiment.

## Conclusion

1. Both types of emotional feedback materials can capture attention, but emotional facial images are more effective at capturing attention than emotional words.
2. When positive and negative information with equal arousal levels alternates over a period, individuals exhibit a stable positive attention bias.
3. When there is intense competition for attention and cognitive resources, emotional information is prioritized for processing.

## Supporting information

S1 File Emotional pictures and words information used in the experiment. (DOCX)
