# Supplementary material for: The influence of emotional feedback material type on attentional capture at different presentation times
Source: PLoS One. 2024 Sep 16;19(9):e0310022. doi: 10.1371/journal.pone.0310022 (PMC11404810; doi:10.1371/journal.pone.0310022)
Supplement: S1 File — (DOCX) [file pone.0310022.s001.docx]

**S1 File. Emotional pictures and words information**

**used in the experiment**

**1 Revised Chinese Facial Affective Picture System (CFAPS) (Gong et al., 2011)**

As this is a system that needs to be purchased. We have only acquired the rights to use the system and therefore cannot provide the original images directly in the supplementary material. If any reader needs this system, please contact the original author.

Selected Affective Picture:

| **No** | **Type** | **Pleasantness** | **Arousal degree** |
| --- | --- | --- | --- |
| NF81 | Negative | 3.61 | 6.33 |
| NF85 | Negative | 3.5 | 6.33 |
| NF34 | Negative | 3.45 | 6 |
| NF82 | Negative | 4.27 | 3.33 |
| NF94 | Negative | 3.39 | 7 |
| NF18 | Negative | 3.11 | 7.33 |
| NF37 | Negative | 2.98 | 6.33 |
| NF83 | Negative | 3.05 | 7 |
| NF70 | Negative | 3.57 | 4.33 |
| NF66 | Negative | 3.55 | 4.67 |
| NF93 | Negative | 3.52 | 7 |
| NF40 | Negative | 2.2 | 7 |
| NF57 | Negative | 2.34 | 7.67 |
| NF53 | Negative | 2.11 | 6.67 |
| NF52 | Negative | 3.02 | 6 |
| NF33 | Negative | 2.32 | 7 |
| NF32 | Negative | 4.43 | 7.33 |
| NF45 | Negative | 2.93 | 5.67 |
| NF79 | Negative | 3.57 | 3.33 |
| NF11 | Negative | 2.7 | 5.67 |
| NM81 | Negative | 3.07 | 7.67 |
| NM85 | Negative | 4.09 | 7.33 |
| NM34 | Negative | 2.68 | 5.67 |
| NM82 | Negative | 3.11 | 6.67 |
| NM94 | Negative | 2.77 | 7 |
| NM18 | Negative | 2.55 | 6.33 |
| NM37 | Negative | 2.14 | 7.67 |
| NM83 | Negative | 3.86 | 8.33 |
| NM70 | Negative | 2.68 | 7 |
| NM66 | Negative | 2.64 | 7 |
| NM93 | Negative | 2.73 | 7.67 |
| NM40 | Negative | 3.32 | 6.33 |
| NM57 | Negative | 2.75 | 6 |
| NM53 | Negative | 2.82 | 6.67 |
| NM52 | Negative | 3.34 | 5.33 |
| NM33 | Negative | 2.34 | 6 |
| NM32 | Negative | 3.09 | 5.33 |
| NM45 | Negative | 2.52 | 8 |
| NM79 | Negative | 2.23 | 5.67 |
| NM11 | Negative | 3.07 | 4 |
| TF81 | Neutral | 4.05 | 3.67 |
| TF85 | Neutral | 4.82 | 4 |
| TF34 | Neutral | 4.93 | 3.33 |
| TF82 | Neutral | 4.3 | 4.33 |
| TF94 | Neutral | 5 | 5 |
| TF18 | Neutral | 4.02 | 4.33 |
| TF37 | Neutral | 4.5 | 3.67 |
| TF83 | Neutral | 4.43 | 4.67 |
| TF70 | Neutral | 4.75 | 4.33 |
| TF66 | Neutral | 4.48 | 3.67 |
| TF93 | Neutral | 5.05 | 3.33 |
| TF40 | Neutral | 4.57 | 4.33 |
| TF57 | Neutral | 4.25 | 4.67 |
| TF53 | Neutral | 4.43 | 5 |
| TF52 | Neutral | 4.7 | 4.67 |
| TF33 | Neutral | 3.45 | 4.67 |
| TF32 | Neutral | 4.34 | 3.33 |
| TF45 | Neutral | 4.14 | 5 |
| TF79 | Neutral | 4.86 | 3.67 |
| TF11 | Neutral | 4.45 | 4.67 |
| TM81 | Neutral | 4.5 | 4.33 |
| TM85 | Neutral | 3.93 | 3.67 |
| TM34 | Neutral | 4.05 | 3.67 |
| TM82 | Neutral | 5.5 | 4.33 |
| TM94 | Neutral | 2.89 | 3.67 |
| TM18 | Neutral | 5.34 | 3 |
| TM37 | Neutral | 3.8 | 3 |
| TM83 | Neutral | 4.05 | 3.67 |
| TM70 | Neutral | 4.3 | 3.33 |
| TM66 | Neutral | 4.64 | 3.67 |
| TM93 | Neutral | 4.48 | 4 |
| TM40 | Neutral | 3.89 | 3.33 |
| TM57 | Neutral | 4.14 | 4 |
| TM53 | Neutral | 4.32 | 2 |
| TM52 | Neutral | 3.59 | 5 |
| TM33 | Neutral | 4.05 | 3.33 |
| TM32 | Neutral | 3.16 | 3.33 |
| TM45 | Neutral | 4.23 | 3 |
| TM79 | Neutral | 3.91 | 3.33 |
| TM11 | Neutral | 3.75 | 3.33 |
| PF81 | Positive | 5.59 | 5 |
| PF85 | Positive | 5.59 | 4.33 |
| PF34 | Positive | 6.07 | 4.67 |
| PF82 | Positive | 6.5 | 5.67 |
| PF94 | Positive | 6.57 | 5 |
| PF18 | Positive | 5.45 | 4.33 |
| PF37 | Positive | 4.82 | 4.67 |
| PF83 | Positive | 4.84 | 2.67 |
| PF70 | Positive | 6.39 | 4.67 |
| PF66 | Positive | 5.66 | 5.33 |
| PF93 | Positive | 6.02 | 6 |
| PF40 | Positive | 6.55 | 5.33 |
| PF57 | Positive | 6.68 | 5 |
| PF53 | Positive | 5.18 | 5.67 |
| PF52 | Positive | 5.5 | 4.67 |
| PF33 | Positive | 6.02 | 7 |
| PF32 | Positive | 5.55 | 4.67 |
| PF45 | Positive | 6.16 | 5 |
| PF79 | Positive | 5.98 | 3.33 |
| PF11 | Positive | 7.14 | 5.33 |
| PM81 | Positive | 5.39 | 5 |
| PM85 | Positive | 6.02 | 4.33 |
| PM34 | Positive | 6.11 | 5.33 |
| PM82 | Positive | 5.86 | 6 |
| PM94 | Positive | 7.02 | 7 |
| PM18 | Positive | 5.75 | 4.33 |
| PM37 | Positive | 6.48 | 4.67 |
| PM83 | Positive | 5.93 | 5.33 |
| PM70 | Positive | 5 | 5 |
| PM66 | Positive | 5.11 | 4 |
| PM93 | Positive | 5.59 | 6.67 |
| PM40 | Positive | 6.64 | 5.67 |
| PM57 | Positive | 5.39 | 5 |
| PM53 | Positive | 6.93 | 5 |
| PM52 | Positive | 6.59 | 7 |
| PM33 | Positive | 5.39 | 3.67 |
| PM32 | Positive | 5.39 | 6.33 |
| PM45 | Positive | 5.25 | 4.33 |
| PM79 | Positive | 5.34 | 4.67 |
| PM11 | Positive | 5.98 | 4 |

1. **Chinese affective words system (CAWS) (Wang et al., 2008)**

Selected emotional words:

| **No** | **Word** | **Type** | **Valence** | **Arousal degree** |
| --- | --- | --- | --- | --- |
| 1 | 无能 | Negative | 2.80 | 4.40 |
| 2 | 懈怠 | Negative | 2.89 | 4.12 |
| 3 | 衰老 | Negative | 2.93 | 4.65 |
| 4 | 处罚 | Negative | 2.94 | 5.23 |
| 5 | 故障 | Negative | 2.96 | 4.84 |
| 6 | 乞丐 | Negative | 2.98 | 4.70 |
| 7 | 奴才 | Negative | 2.98 | 4.70 |
| 8 | 懒惰 | Negative | 3.03 | 4.34 |
| 9 | 倒闭 | Negative | 3.11 | 5.54 |
| 10 | 妨碍 | Negative | 3.13 | 4.72 |
| 11 | 昏暗 | Negative | 3.18 | 4.55 |
| 12 | 失意 | Negative | 3.18 | 4.68 |
| 13 | 孤立 | Negative | 3.22 | 4.85 |
| 14 | 阴影 | Negative | 3.22 | 4.34 |
| 15 | 丢失 | Negative | 3.24 | 5.21 |
| 16 | 病房 | Negative | 3.24 | 4.70 |
| 17 | 恍惚 | Negative | 3.29 | 4.12 |
| 18 | 别扭 | Negative | 3.33 | 4.84 |
| 19 | 僵硬 | Negative | 3.34 | 4.05 |
| 20 | 衰退 | Negative | 3.35 | 4.40 |
| 21 | 废弃 | Negative | 3.40 | 4.40 |
| 22 | 糊涂 | Negative | 3.42 | 4.05 |
| 23 | 厌倦 | Negative | 3.43 | 4.65 |
| 24 | 废话 | Negative | 3.43 | 4.42 |
| 25 | 虚弱 | Negative | 3.46 | 4.05 |
| 26 | 气喘 | Negative | 3.53 | 4.42 |
| 27 | 沉痛 | Negative | 3.61 | 5.54 |
| 28 | 否认 | Negative | 3.70 | 4.67 |
| 29 | 包袱 | Negative | 3.76 | 4.40 |
| 30 | 是非 | Negative | 3.83 | 4.85 |
| 31 | 阻拦 | Negative | 3.83 | 4.72 |
| 32 | 要害 | Negative | 3.84 | 5.54 |
| 33 | 兵器 | Negative | 3.88 | 5.23 |
| 34 | 惭愧 | Negative | 3.93 | 4.72 |
| 35 | 解散 | Negative | 3.93 | 4.65 |
| 36 | 迷惑 | Negative | 3.95 | 4.84 |
| 37 | 牲畜 | Negative | 3.98 | 4.12 |
| 38 | 引诱 | Negative | 4.00 | 5.54 |
| 39 | 插嘴 | Negative | 4.08 | 4.68 |
| 40 | 审查 | Negative | 4.13 | 4.72 |
| 41 | 顾虑 | Neutral | 4.22 | 4.70 |
| 42 | 行踪 | Neutral | 4.29 | 4.40 |
| 43 | 占据 | Neutral | 4.35 | 4.65 |
| 44 | 苦心 | Neutral | 4.37 | 4.48 |
| 45 | 奔波 | Neutral | 4.37 | 5.23 |
| 46 | 怜惜 | Neutral | 4.63 | 4.40 |
| 47 | 脸色 | Neutral | 4.78 | 4.42 |
| 48 | 警察 | Neutral | 4.80 | 4.84 |
| 49 | 沉醉 | Neutral | 4.87 | 4.42 |
| 50 | 厉害 | Neutral | 4.90 | 5.54 |
| 51 | 化装 | Neutral | 4.96 | 4.42 |
| 52 | 法制 | Neutral | 5.00 | 4.68 |
| 53 | 开支 | Neutral | 5.11 | 4.55 |
| 54 | 履行 | Neutral | 5.12 | 4.40 |
| 55 | 掩护 | Neutral | 5.16 | 4.42 |
| 56 | 发掘 | Neutral | 5.19 | 4.84 |
| 57 | 辩护 | Neutral | 5.20 | 5.23 |
| 58 | 消遣 | Neutral | 5.22 | 4.42 |
| 59 | 贵族 | Neutral | 5.29 | 4.85 |
| 60 | 淘气 | Neutral | 5.37 | 4.70 |
| 61 | 军官 | Neutral | 5.37 | 4.55 |
| 62 | 挽救 | Neutral | 5.41 | 5.21 |
| 63 | 锐利 | Neutral | 5.47 | 4.70 |
| 64 | 支配 | Neutral | 5.50 | 4.34 |
| 65 | 干部 | Neutral | 5.54 | 4.34 |
| 66 | 经费 | Neutral | 5.55 | 4.68 |
| 67 | 严密 | Neutral | 5.56 | 4.48 |
| 68 | 首领 | Neutral | 5.65 | 5.21 |
| 69 | 筹划 | Neutral | 5.68 | 4.42 |
| 70 | 证实 | Neutral | 5.72 | 4.65 |
| 71 | 储蓄 | Neutral | 5.80 | 4.42 |
| 72 | 和解 | Neutral | 5.83 | 4.70 |
| 73 | 协作 | Neutral | 5.87 | 4.48 |
| 74 | 地位 | Neutral | 5.89 | 5.21 |
| 75 | 竣工 | Neutral | 5.92 | 4.85 |
| 76 | 有效 | Neutral | 5.98 | 4.55 |
| 77 | 飞行 | Neutral | 6.03 | 4.72 |
| 78 | 情调 | Neutral | 6.12 | 4.55 |
| 79 | 气派 | Neutral | 6.15 | 5.23 |
| 80 | 高速 | Neutral | 6.16 | 4.70 |
| 81 | 形象 | Positive | 6.19 | 4.72 |
| 82 | 敏捷 | Positive | 6.21 | 4.70 |
| 83 | 亲近 | Positive | 6.22 | 4.85 |
| 84 | 赞成 | Positive | 6.22 | 4.85 |
| 85 | 情感 | Positive | 6.23 | 4.68 |
| 86 | 锻炼 | Positive | 6.26 | 4.65 |
| 87 | 期待 | Positive | 6.26 | 4.48 |
| 88 | 家长 | Positive | 6.29 | 4.72 |
| 89 | 鲜明 | Positive | 6.29 | 4.70 |
| 90 | 精神 | Positive | 6.30 | 4.68 |
| 91 | 亲戚 | Positive | 6.30 | 4.65 |
| 92 | 好转 | Positive | 6.30 | 4.55 |
| 93 | 典礼 | Positive | 6.32 | 4.85 |
| 94 | 营养 | Positive | 6.38 | 4.48 |
| 95 | 本领 | Positive | 6.41 | 4.84 |
| 96 | 心情 | Positive | 6.43 | 4.72 |
| 97 | 漫画 | Positive | 6.45 | 4.55 |
| 98 | 科学 | Positive | 6.54 | 4.72 |
| 99 | 滑雪 | Positive | 6.55 | 4.65 |
| 100 | 准时 | Positive | 6.56 | 4.48 |
| 101 | 新颖 | Positive | 6.57 | 4.84 |
| 102 | 用功 | Positive | 6.60 | 4.72 |
| 103 | 生动 | Positive | 6.62 | 4.85 |
| 104 | 榜样 | Positive | 6.63 | 4.85 |
| 105 | 真实 | Positive | 6.67 | 4.65 |
| 106 | 有利 | Positive | 6.70 | 4.85 |
| 107 | 清秀 | Positive | 6.70 | 4.48 |
| 108 | 提高 | Positive | 6.71 | 4.48 |
| 109 | 气质 | Positive | 6.74 | 4.68 |
| 110 | 真理 | Positive | 6.78 | 4.85 |
| 111 | 公平 | Positive | 6.83 | 4.48 |
| 112 | 俊秀 | Positive | 6.83 | 4.68 |
| 113 | 兄弟 | Positive | 6.84 | 4.55 |
| 114 | 尊重 | Positive | 6.87 | 4.85 |
| 115 | 进步 | Positive | 6.88 | 4.68 |
| 116 | 芳香 | Positive | 6.91 | 4.55 |
| 117 | 廉洁 | Positive | 6.93 | 4.70 |
| 118 | 优点 | Positive | 6.95 | 4.84 |
| 119 | 家庭 | Positive | 7.03 | 4.84 |
| 120 | 笑容 | Positive | 7.23 | 4.65 |
